# Supplementary material for: Fecal Volatile Organic Compounds and Microbiota Associated with the Progression of Cognitive Impairment in Alzheimer’s Disease
Source: Int J Mol Sci. 2022 Dec 31;24(1):707. doi: 10.3390/ijms24010707 (PMC9821163; doi:10.3390/ijms24010707)
Supplement: Supplementary file 1 [file ijms-24-00707-s001.zip › Supplementary Figure S1.pptx]

## Slide 1
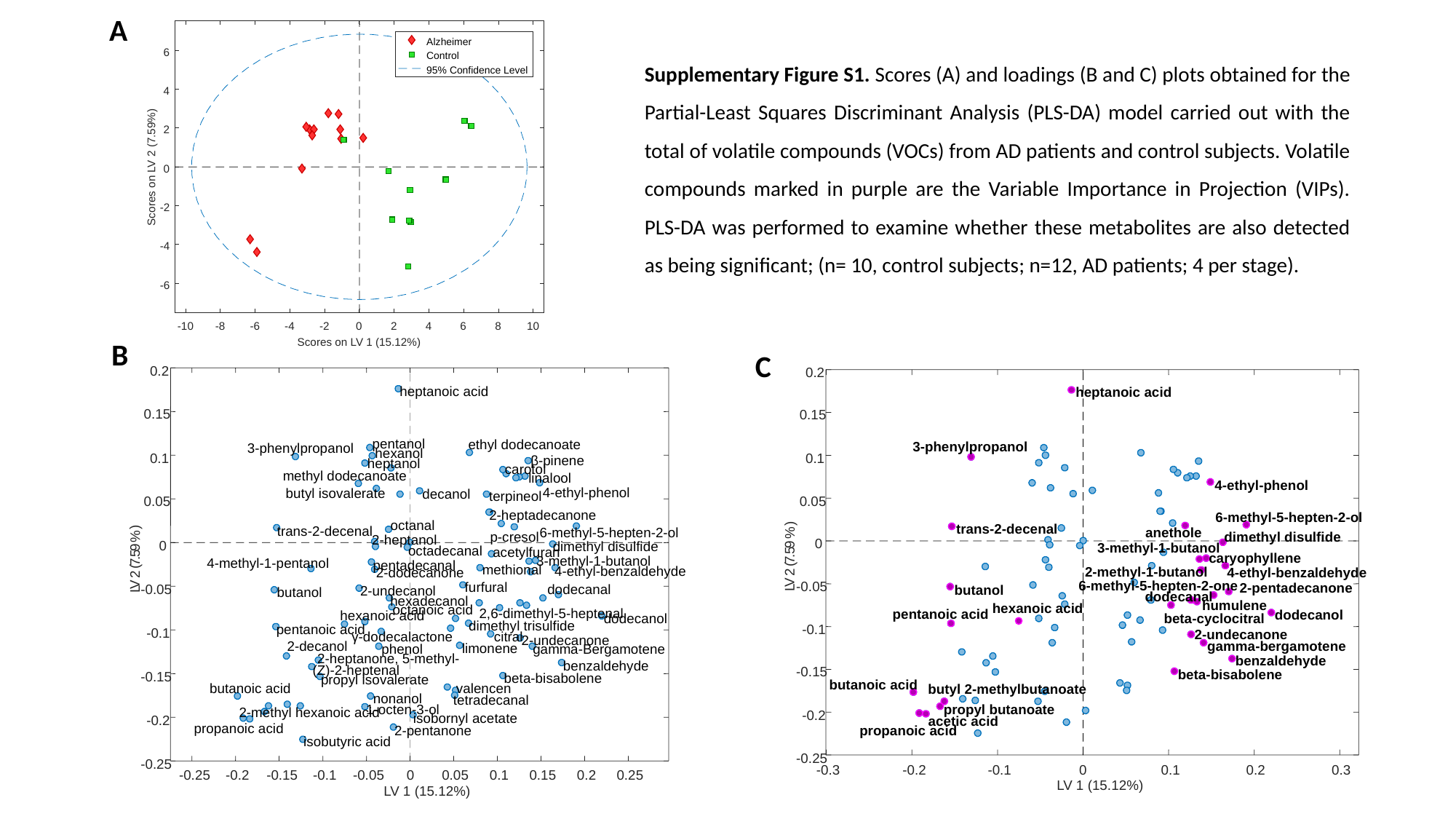

A
Supplementary Figure S1. Scores (A) and loadings (B and C) plots obtained for the Partial-Least Squares Discriminant Analysis (PLS-DA) model carried out with the total of volatile compounds (VOCs) from AD patients and control subjects. Volatile compounds marked in purple are the Variable Importance in Projection (VIPs). PLS-DA was performed to examine whether these metabolites are also detected as being significant; (n= 10, control subjects; n=12, AD patients; 4 per stage).
B
0.2
0.15
0.1
0.05
)
%
0
9
5
.
7
(
2
V
-0.05
L
-0.1
-0.15
-0.2
-0.25
-0.25
-0.2
-0.15
-0.1
-0.05
0
0.05
0.1
0.15
0.2
0.25
LV 1 (15.12%)
 heptanoic acid
 pentanol
 ethyl dodecanoate
 3-phenylpropanol
 hexanol
 β-pinene
 heptanol
 carotol
 methyl dodecanoate
 linalool
 4-ethyl-phenol
 butyl isovalerate
 decanol
 terpineol
 2-heptadecanone
 octanal
 trans-2-decenal
 6-methyl-5-hepten-2-ol
 p-cresol
 2-heptanol
 dimethyl disulfide
 octadecanal
 acetylfuran
 3-methyl-1-butanol
 4-methyl-1-pentanol
 pentadecanal
 methional
 4-ethyl-benzaldehyde
 2-dodecanone
 furfural
 dodecanal
 2-undecanol
 butanol
 hexadecanol
 octanoic acid
 2,6-dimethyl-5-heptenal
 hexanoic acid
 dodecanol
 dimethyl trisulfide
 pentanoic acid
γ-dodecalactone
 citral
 2-undecanone
 2-decanol
 limonene
 phenol
 gamma-Bergamotene
 2-heptanone, 5-methyl-
 benzaldehyde
 (Z)-2-heptenal
 beta-bisabolene
 propyl isovalerate
 butanoic acid
 valencen
 nonanol
 tetradecanal
 1-octen-3-ol
 2-methyl hexanoic acid
 isobornyl acetate
 propanoic acid
 2-pentanone
 isobutyric acid
0.2
0.15
0.1
0.05
)
%
0
9
5
.
7
(
2
V
-0.05
L
-0.1
-0.15
-0.2
-0.25
-0.3
-0.2
-0.1
0
0.1
0.2
0.3
LV 1 (15.12%)
 heptanoic acid
 3-phenylpropanol
 4-ethyl-phenol
 6-methyl-5-hepten-2-ol
 trans-2-decenal
 anethole
 dimethyl disulfide
 3-methyl-1-butanol
 caryophyllene
 2-methyl-1-butanol
 4-ethyl-benzaldehyde
 6-methyl-5-hepten-2-one
 2-pentadecanone
 butanol
 dodecanal
 humulene
 pentanoic acid
 dodecanol
beta-cyclocitral
 2-undecanone
 gamma-bergamotene
 benzaldehyde
 beta-bisabolene
 butanoic acid
 butyl 2-methylbutanoate
 propyl butanoate
 acetic acid
 propanoic acid
C
 hexanoic acid
