# Supplementary material for: Fecal Volatile Organic Compounds and Microbiota Associated with the Progression of Cognitive Impairment in Alzheimer’s Disease
Source: Int J Mol Sci. 2022 Dec 31;24(1):707. doi: 10.3390/ijms24010707 (PMC9821163; doi:10.3390/ijms24010707)
Supplement: Supplementary file 1 [file ijms-24-00707-s001.zip › Tabla S1.pdf]

Table S1. Volatile organic compounds (VOCs) relative abundance in Alzheimer´s disease (AD) patients and healthy controls.

VOCS	Control	AD	P
Terpenes			
alpha pinene	79.56±54.5	7.14±3.2	<0.0001
beta-pinene	108.78±70.5	15.96±8.4	<0.0001
beta myrcene	1.28±1.04	0.07±0.07	ns
3-carene	189.47±152.7	5.84±5.3	<0.0001
phellandrene	4.84±3.2	0.04±0.02	ns
limonene	365.03±159.2	167.677.6	<0.0001
gamma-terpinene	41.1±18.4	18.8±9.2	<0.05
linalool	15.53±13.1	0.16±0.11	<0.05
gamma-Bergamotene	1.28±0.47	0.09±0.08	ns
caryophyllene	8.21±4.1	1.46±0.5	ns
menthol	2.15±0.9	1.26±0.4	ns
beta-cyclocitral	1.57±0.6	0.39±0.26	ns
cis-beta-Farnesene	0.52±0.4	3.89±3.7	ns
humulene	1.91±1	0±0	ns
terpineol	1.22±1	0.42±0.22	ns
valencen	24.7±14.4	8.47±5.4	<0.05
beta-bisabolene	0.21±0.1	0.01±0.01	ns
citral	6.39±1.9	3.69±1	ns
anethole	0.14±0.1	0±0	ns
trans-calamenene	12.61±8.2	35.58±32.6	<0.05
carotol	0.1±0.1	0±0	ns
Ketones			
2-pentanone	53.11±19.2	46.5±11.4	ns
5-methyl-2-heptanone	3.2±1.6	6.5±1.6	ns
6-methyl-5-hepten-2-one	121.42±24.1	70.36±16	<0.05
acetylfuran	2.88±0.7	1.85±0.24	ns
2-undecanone	10.62±1.3	6.87±1	ns
2-dodecanone	2.65±0.4	3.71±1.8	ns
2-pentadecanone	10.14±1.8	4.44±0.9	ns
2-heptadecanone	0.66±0.2	0.36±0.27	ns
Sulfur compounds			
dimethyl disulfide	178.88±61.4	23.43±7.6	<0.0001
dimethyl trisulfide	37.67±22.9	9.65±5.5	<0.05
Esters			
propyl butanoate	0.32±0.3	41.68±21.7	<0.05
propyl isovalerate	0.48±0.3	6.65±6	ns
butyl 2-methylbutanoate	1.07±0.6	17.49±8.7	<0.05
butyl isovalerate	0±0	2.18±1.7	ns
isobornyl acetate	0.24±0.15	0.11±0.09	ns
methyl salicylate	0.47±0.45	2.04±1	ns
methyl dodecanoate	0.08±0.08	0.3±0.3	ns
ethyl dodecanoate	0.08±0.07	0.04±0.04	ns

gamma-nonalactone	3.47±0.9	2.45±0.25	ns
gamma-dodecalactone	15.68±3.8	18.58±2	ns
Alcohols			
butanol	6.48±3.5	37.57±11.9	<0.0001
2-methyl-1-butanol	29.8±11.6	5.95±1.9	<0.05
3-methyl-1-butanol	28.95±11.2	6.08±1.5	<0.05
pentanol	8.52±4.3	16.27±4.4	ns
4-methyl-1-pentanol	0.69±0.5	1.82±0.6	ns
2-heptanol	4.06±0.7	5.27±1.6	ns
hexanol	11.14±4.9	28.8±16.3	<0.05
1-octen-3-ol	9.60±2.2	10.05±3.9	ns
heptanol	1.62±0.85	11.86±9	ns
6-methyl-5-hepten-2-ol	14.35±4.2	2.23±0.8	ns
2-decanol	0.75±0.3	1.67±0.5	ns
nonanol	17.75±12	19.52±9.5	ns
2-undecanol	2.94±0.7	3.5±0.8	ns
decanol	0.18±0.11	0.18±0.17	ns
phenylethyl alcohol	21.18±4	26.86±5.2	ns
dodecanol	21.20±4	3.77±0.8	<0.05
phenol	174.73±134.9	203.6±76.3	ns
3-phenylpropanol	0.29±0.14	2.96±1	ns
p-cresol	1105.94±162.9	927.8±107.6	<0.05
4-ethyl-phenol	4.68±1.9	1.37±0.26	<0.05
hexadecanol	0.88±0.7	1.01±0.26	ns
Aldehydes			
octanal	0.84±0.2	1.1±0.4	ns
(Z)-2-heptenal	0.98±0.3	3.1±1.7	ns
2.6-dimethyl-5-heptenal	1.55±0.5	0.88±0.3	ns
methional	4.18±2.1	2.13±0.9	ns
furfural	30.49±23.5	2.05±0.7	<0.05
benzaldehyde	124.65±27.7	39.06±5.2	<0.0001
trans-2-decenal	2.17±0.9	24.65±7.6	<0.05
dodecanal	3.59±0.9	1.51±0.3	ns
4-ethyl-benzaldehyde	0.14±0.01	0.11±0.01	ns
tetradecanal	3.95±1.5	2.64±0.8	ns
pentadecanal	2.52±0.7	3.51±0.8	ns
octadecanal	2±0.8	2.33±0.8	ns
Acids			
acetic acid	16.68±7.6	69.55±22	<0.05
propanoic acid	23.41±11.5	144.71±48	<0.0001
isobutyric acid	30.14±10.3	46.46±10.2	ns
butanoic acid	141.3±59.2	646.57±177	<0.0001
isovaleric acid	101.59±36.5	138.01±33.5	ns
2-methyl hexanoic acid	37.26±6.2	59.11±11.4	<0.05
pentanoic acid	143.84±66.4	425.8±108.7	<0.0001
hexanoic acid	118.06±82	218.41±90	<0.0001
heptanoic acid	12.48±9	513±25	<0.05

octanoic acid	17.01±11.4	13.83±8	ns

Fecal VOCs relative abundance from healthy subjects and all AD patients grouped. Results are presented as means ± SEM; (n= 10, control subjects; n=12, AD patients). Comparisons were performed with ANOVA followed by Tukey´s test; ns, not significant.
