# Supplementary material for: Fecal Volatile Organic Compounds and Microbiota Associated with the Progression of Cognitive Impairment in Alzheimer’s Disease
Source: Int J Mol Sci. 2022 Dec 31;24(1):707. doi: 10.3390/ijms24010707 (PMC9821163; doi:10.3390/ijms24010707)
Supplement: Supplementary file 1 [file ijms-24-00707-s001.zip › Table S2.pdf]

Table S2. Relative abundance of OTUs in Alzheimer´s disease (AD) patients and healthy controls.

**OTUs**	**Control**	**AD**	**P**
p__Actinobacteriota	5015.1 ± 2308	3277.8 ± 1301	<0.0001
p__Bacteroidota	5713.2 ± 1422	8921.5 ± 1725	<0.0001
p__Firmicutes	47997.8 ± 4382	45167.4 ± 3042	<0.0001
c__Actinobacteria	4257.9 ± 2255	2394.9 ± 1240	<0.01
c__Bacteroidia	5713.2 ± 1422	8921.6 ± 1726	<0.0001
c__Clostridia	43868.9 ± 3825	39745±3449	<0.0001
f__Bifidobacteriaceae	4252±2254	2389±1239	<0.0001
f__Bacteroidaceae	4075.7±1345	6011.1±1523	<0.0001
f__Veillonellaceae	1452.5±795	371.6±226	<0.05
f__Akkermansiaceae	797.4±283	1061.9±439	<0.01
g__Bifidobacterium	4252±2254	2382.7±1240	<0.0001
g__Bacteroides	4075.71345	6011.1±1523	<0.0001
g__Barnesiella	94.7±56	234.8±51	ns
g__Alistipes	404.9±85	952.8±285	<0.0001
g__Clostridium	3461.5±1263	752.4±216	<0.0001
g__Blautia	2287.4±368	3614.9±775	<0.0001
g__Coprococcus	1783.8±517	940.5±382	<0.0001
g__Lachnoclostridium	219.9±45	659.3±249	<0.0001
g__Roseburia	454.2±93	1445.9±362	<0.0001
g__Faecalibacterium	9273.6±1644	8713.5±2825	<0.0001
g__Ruminococcus	1039.1±1039	1408.1±746	<0.0001
g__Peptococcus	11.3±5	44.9±20	ns
g__Akkermansia	797.4±283	1061.9±439	<0.001
s_Akkermansia_muciniphila	665±302	978±448	<0.0001

Gut bacterial taxa relative abundance of fecal samples from control subjects and all AD patients grouped. p, phylum; c, class; f, family; g, genus; s, species. Results are presented as means ± SEM; (n= 10, control subjects; n=12, AD patients). Comparisons were performed with ANOVA followed by Tukey´s test; ns, not significant.
